# Transcriptome Analysis to Shed Light on the Molecular Mechanisms of Early Responses to Cadmium in Roots and Leaves of King Grass (*Pennisetum americanum* × *P. purpureum*)

**DOI:** 10.3390/ijms20102532

**Published:** 2019-05-23

**Authors:** Junming Zhao, Bo Xia, Yu Meng, Zhongfu Yang, Ling Pan, Man Zhou, Xinquan Zhang

**Affiliations:** 1 Department of Grassland Science, Sichuan Agricultural University, Chengdu 611130, China; junmingzhao163@163.com (J.Z.); yangzf211@163.com (Z.Y.); panling1199@126.com (L.P.); 2 Department of Genetics and Biochemistry, Clemson University, Clemson, SC 29634-0318, USA; bxia@g.clemson.edu; 3 College of Natural, Applied and Health Sciences, Wenzhou Kean University, Wenzhou 325060, China; meng.yoyo@wku.edu.cn

**Keywords:** *Pennisetum americanum* × *P. purpureum*, transcriptome, differentially expressed genes, Cd responses, molecular mechanism

## Abstract

King grass, a hybrid grass between pearl millet and elephant grass, has many excellent characteristics such as high biomass yield, great stress tolerance, and enormous economic and ecological value, which makes it ideal for development of phytoremediation. At present, the physiological and molecular response of king grass to cadmium (Cd) stress is poorly understood. Transcriptome analysis of early response (3 h and 24 h) of king grass leaves and roots to high level Cd (100 µM) has been investigated and has shed light on the molecular mechanism underlying Cd stress response in this hybrid grass. Our comparative transcriptome analysis demonstrated that in combat with Cd stress, king grass roots have activated the glutathione metabolism pathway by up-regulating glutathione S-transferases (GSTs) which are a multifunctional family of phase II enzymes that detoxify a variety of environmental chemicals, reactive intermediates, and secondary products of oxidative damages. In roots, early inductions of phenylpropanoid biosynthesis and phenylalanine metabolism pathways were observed to be enriched in differentially expressed genes (DEGs). Meanwhile, oxidoreductase activities were significantly enriched in the first 3 h to bestow the plant cells with resistance to oxidative stress. We also found that transporter activities and jasmonic acid (JA)-signaling might be activated by Cd in king grass. Our study provided the first-hand information on genome-wide transcriptome profiling of king grass and novel insights on phytoremediation.

## 1. Introduction

Cadmium (Cd) is one of the most hazardous heavy metal pollutants that is massively released from human activities which can induce amounts of reactive oxygen species (ROSs) and have negative effects on protein, nucleic acids, gene expressions, and some essential enzymatic activities causing toxicity and mutagenesis in cells [1]. Cd accumulates in plants mostly by soil absorption and can be transferred to other organisms by food chain. The consumption of Cd-contaminated food threatens human health worldwide [2,3]. Under such context, it is of urgent importance to develop methods to clean up contaminated soil. Phytoremediation—the use of plants to absorb heavy metal from soil, is one of the most promising technologies to solve this problem [4]. The plants which are suitable for phytoremediation in general have the following characteristics: (1) fast growth rate, (2) high biomass yields, (3) and can be easily harvested with high accumulation of a range of heavy metal in harvestable organs [5]. From molecular perspectives, a good candidate for phytoremediation, in other words a hyper-accumulator, should have an efficient root uptake genetic system, as well as sets of robust genes responsible for root-to-shoot translocation of metals.

*Pennisetum americanum* × *P. purpureum*, also named king grass, is a perennial grass species which belongs to the genus *Pennisetum.* It is a hybrid between pearl millet and elephant grass which combines the good qualities of both. Its roots are deep, dense and highly developed. It also has a fast growth rate and high regeneration ability. The stems are round and erect, and the plant shape is compact. The plant height is around 3.5 m, yet sometimes it can reach more than 4 m [6]. King grass serves as a good energy grass and ornamental grass with excellent characteristics such as drought tolerance, flooding tolerance, heat tolerance, disease resistance, and lodging tolerance [7]. King grass is mainly planted in the Yangzi River basin and south of the Huaihe River in China and other tropical and subtropical regions globally [8]. Plus, this hybrid grass has high biomass yield, broad ecological merits, and enormous economic values, which makes it ideal to be developed for phytoremediation.

So far, we have already accumulated some knowledge about the molecular mechanisms underlying heavy metal absorption, transportation, and relocation in herbaceous model plant species. For example, in both *Brassica napus* and its close relative *Arabidopsis*, besides the well-known mechanism that phytochelatins (PCs) and glutathiones (GSHs) can bind to Cd to form Cd-ligands and then be shut off in vacuoles serving as a detoxification tool, it is also proposed that PCs and GSHs function as long distance carriers between roots and shoots [9]. In *Arabidopsis*, metallothioneins (MTs) and PCs work together to protect plants from the toxicity of copper (Cu) and Cd [10]. Several metal transporter genes such as *ZIP9*, *ZIP6*, and *ZIP3* are indicated to be highly involved in cytoplasmic Cd influx or from the root symplast into the xylem apoplast [11]. In addition, it is confirmed that Zinc transport system can be used for Cd transport, and Cd is translocated from roots to shoots through xylem transport as free ions instead of forming complex with citrate in *Arabidopsis halleri* [12]. Since we are interested in applying king grass or other related grass species in phytoremediation, it is essential to investigate the mechanisms underlying king grass Cd response using a broad scale approach such as transcriptome profiling.

Recently, several studies focused on the effects of Cd on specific organs such as rice roots and leaves [13], hybrid poplars bark [14], *Arabidopsis* roots and leaves [15], *Salix matsudana* Koidz roots and leaves [5], and pea leaves [16]. However, the Cd effects on transcriptional and organ-specific level in king grass remains unknown. In this study, we irrigated the roots of the king grass with heavy metal CdCl_2_ solution, and the samples of roots and leaves at 0 h (T0), 3 h (T3), and 24 h (T24) were then harvested, with three replicates for each organ type and time point subjected to RNA-seq. Our comparative transcriptome analysis elucidated the molecular mechanisms of early responses to Cd stress in king grass providing novel perspectives into phytoremediation investigation.

## 2. Results and Discussions

### 2.1. Cd Concentration in Leaves and Roots in a Time-Course Treatment of 100 µM Cd

**Figure 1:**
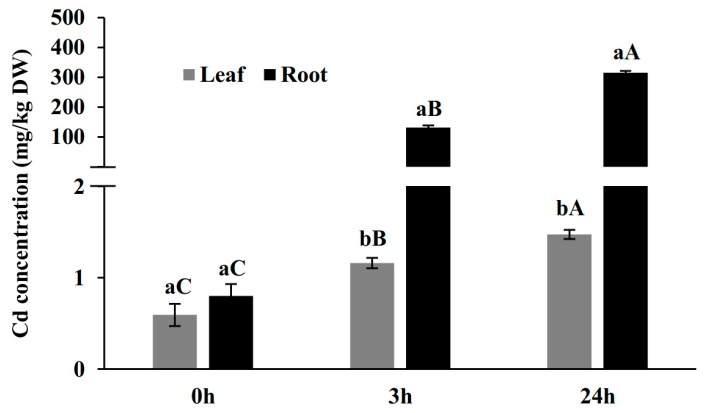
Cadmium (Cd) concentrations in root (black bars) and shoot (gray bars) at T0, T3 and T24. Different small letters indicate significant differences at *p* < 0.05 level of LSD test between two organs at the same time point. Different capital letters indicate significant differences at *p* < 0.05 level among different time points in the same tissue.

Average leaf Cd concentrations at T0, T3, and T24 (0, 3, 24 h after Cd treatment) were 0.59 mg·kg^−1^ dry weight (DW), 1.16 mg·kg^−^ DW, 1.48 mg·kg^−1^ DW, and average root Cd concentration at T0, T3, and T24 were 0.8 mg·kg^−1^ DW, 132 mg·kg^−1^ DW, 315 mg·kg^−1^ DW (Figure 1). The most dramatic change of Cd content occurred at T3 after Cd treatment with the increase of 165 times in roots. Between T3 and T24, the increase in Cd content was still significant (2.4 times comparing to T3 and 394 times comparing to T0) (Figure 1). Meanwhile, the set of data from leaves demonstrated that Cd content increased significantly but gradually from T0 to T3 (1.96 times) and T3 to T24 (2.49 times), but not as dramatically as it occurred in roots (Figure 1). This observation indicated that the early 3 h after Cd treatment was a vital phase for Cd uptake in root and translocation from roots to leaves in response to the Cd stress in king grass at early stage.

### 2.2. RNA Sequencing and Identification of Differentially Expressed Genes (DEGs)

To explore the effect of Cd stress on leaves and roots in a time-course treatment in a molecular level, we conducted an expression profiling experiment using RNA-seq. King grass plant roots were mock-treated (T0) or treated with Cd, and treated samples were collected at T0, T3, and T24. Details of sample preparation and data analysis are provided in materials and methods.

As aforementioned, king grass has no available reference genome sequences. Hence, trinity software was used to *de novo* assemble all the 245, 566, 074 clean reads to generate unique consensus sequences. Totally, there were 365, 298 unigenes yielded from the construct with an N50 unigene size of 900 nt and an average length of 600 nt. About 40% unigenes (146, 650) could be annotated with the reference to the six databases (NR, Swiss-Prot, eggnog, IPR, GO and KEGG). Raw data for this study were deposited at NCBI (National Coalition Building Institute) SAR (Sequence Read Archive) database with the BioProject accession numbers PRJNA521421.

**Figure 2:**
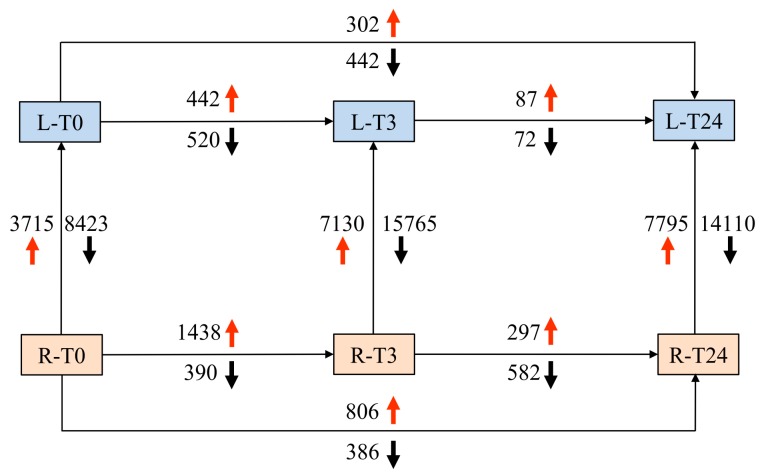
Summary of Differentially Expressed Genes (DEGs) between organs and treatment duration.

With regard to the analysis, the criteria for considering a gene as differentially expressed was a fold-change of 2 or more (|log_2_ fold-change| > 1) and *q* < 0.05. In roots, the expression levels of 1828 genes were altered by Cd treatment at T3 compared to mock-treated plants at T0, with 1438 genes up-regulated (termed as T3 vs. T0 Up or Cd-induced genes at T3 in roots) and 390 genes down-regulated (termed as T3 vs. T0 Down or Cd-repressed genes at T3 in roots) (Figure 2). Comparing to the expression data at T3 vs. T0 and T24 vs. T0, the most remarkable gene expression change occurred during the first 3 h in roots, which is consistent with our Cd concentration measurement data (Figure 1).

In leaves, upon Cd treatment, the expression of 962 genes were altered at T3, 442 of which were up-regulated (termed as T3 vs. T0 Up or Cd-induced genes at T3 in leaves) and 520 of which were down-regulated (termed as T3 vs. T0 Down or Cd-repressed genes at T3 in leaves). Comparing this to the expression data at T3 vs. T0 and T24 vs. T0, in leaves, the most remarkable changes in gene expression also occurred during the first 3 h, which is consistent with our Cd concentration measurement data (Figure 1). Furthermore, by comparing the number of DEGs, gene alternation levels in roots were higher than leaves, indicating that in early responses (Figure 2), much more complicated transcriptional responses occurred in roots.

Due to the different cell types and tissue types composing the two significantly different plant organs, it is natural that roots have significantly different transcriptional profiles at T0 (12, 138 DEGs). However, this difference has been increased to 22, 895 DEGs at T3 and 21, 905 DEGs at T24, which indicated that Cd stress in the first 3 h can significantly impact the transcriptional profiles consistent with our above analysis (Figure 2).

### 2.3. RNA Sequencing Validation by qPCR

To confirm the RNA-seq results, we randomly selected 15 genes that presented up- and down-regulation for qRT-PCR. The relative gene expression of qRT-PCR was calculated by the 2^−ΔΔ^*^C^*^t^ method. The results exhibited a good consistency between the data of RNA-seq and qRT-PCR (r = 0.442, *p* = 0.002) (Figure S1). For each gene, the expression TPM (Transcripts per Million mapped reads) values of transcriptome data displayed similar expression profile at all the three-time point in roots and leaves of king grass compared with the results of qRT-PCR (Figure S2). It showed that a reliable RNA-seq result was obtained in this study.

### 2.4. The Overlaps of DEGs Following CdCl_2_ Exposure at Different Time Points

**Figure 3:**
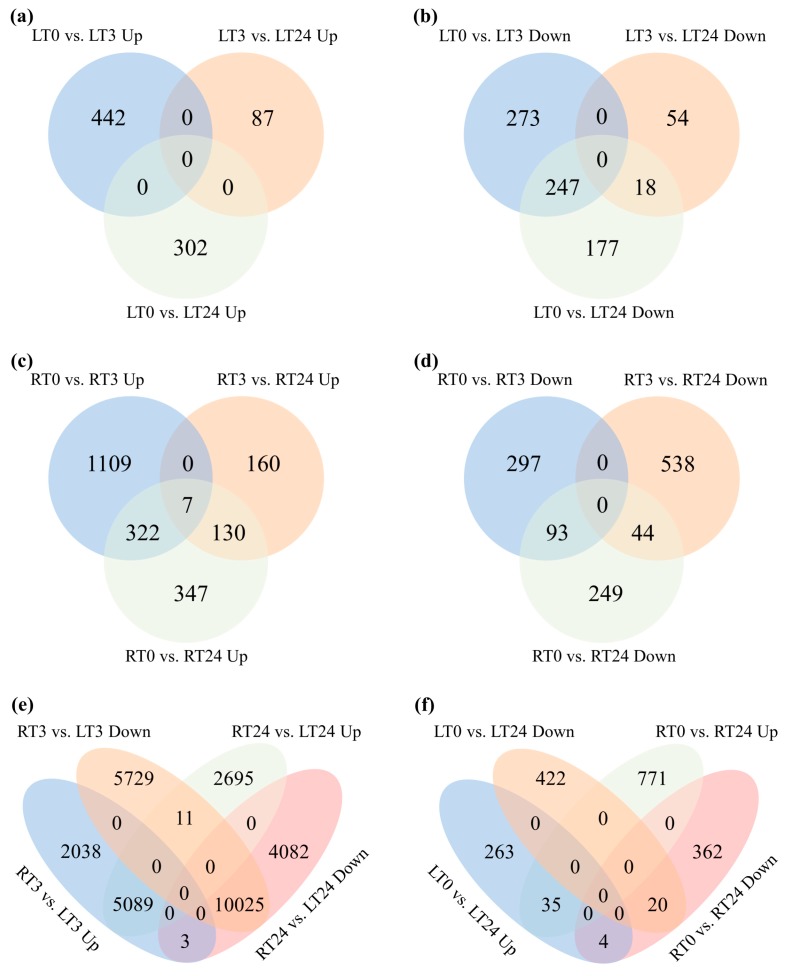
Venn diagrams of differentially expressed genes. Overlaps among up-regulated genes following CdCl_2_ exposure in leaves (**a**), down-regulated genes following CdCl_2_ exposure in leaves (**b**), up-regulated genes following CdCl_2_ exposure in roots (**c**), down-regulated following CdCl_2_ exposure in roots (**d**), up and down-regulated genes following CdCl_2_ exposure at T3 and T24 in organs (**e**), up and down-regulated following CdCl_2_ exposure at T0 and T24 in organs (**f**).

Interestingly, the Cd-induced genes in leaves at T3 and T24 were entirely distinct, indicating that the gene activation was not dominant in first 24 h upon Cd stress (Figure 3a). Among Cd-repressed genes, there were no gene sets which were down-regulated from T0 to T3 and further down-regulated from T3 to T24 as no common gene was found between these two data sets (Figure 3b). However, among Cd-repressed genes, 247 genes negatively responded to the Cd stress during the whole-time course, 18 common genes were down-regulated from T0 to T3 and further down-regulated from T3 to T24 (Figure 3b).

Distinctly different from the response in leaves, in roots the Cd-induced genes at T3 and T24 comparing to T0 had 329 genes in common (Figure 3c), indicating that there were quite a portion of genes being activated in the whole-time course. Seven genes were up-regulated from T0 to T3 and further up-regulated from T3 to T24 (Figure 3c). However, in roots the Cd-repressed genes at T3 and T24 were almost entirely distinct (Figure 3d).

As Cd stress has a profoundly different impact on roots and leaves, we explored the overlapping genes between the leaves and roots at T3 and T24 in both Cd activation and repression modes. Figure 3e demonstrated that 5089 common genes were found between the up-regulated gene sets - RT3 vs. LT3 and RT24 vs. LT24 which represent those up-regulated in roots comparing in leaves during the whole Cd treatment; 10025 common genes were found between the down-regulated gene sets - RT3 vs. LT3 and RT24 vs. LT24 represent those which were down-regulated in roots comparing in leaves during the whole Cd treatment; only **11** common genes were found between the gene sets—RT3 vs. LT3 down and RT24 vs. LT24 up represent those which were down-regulated in roots comparing in leaves at T3 and then up-regulated in roots at T24; only **3** common genes were found between RT3 vs. LT3 up and RT24 vs. LT24 down represent those which were up-regulated in roots at T3 and then down-regulated in roots at T24 comparing in leaves (Figure 3e). Results further indicated that genetic basis in Cd response in leaves and roots were significantly different on early stage.

Although as mentioned above, the genetic basis for Cd response was so distinct, there were slight similarities. Thirty-five common genes were found between the up-regulated gene sets—RT0 vs. LT0 and RT24 vs. LT24 and 20 common genes were found between the down-regulated gene sets – RT0 vs. LT0 and RT24 vs. LT24 represent those which were respond in similar ways in both organs 24 h after Cd treatment (Figure 3f).

### 2.5. Gene Expression Trend Analysis, and GO and KEGG Classification

**Figure 4:**
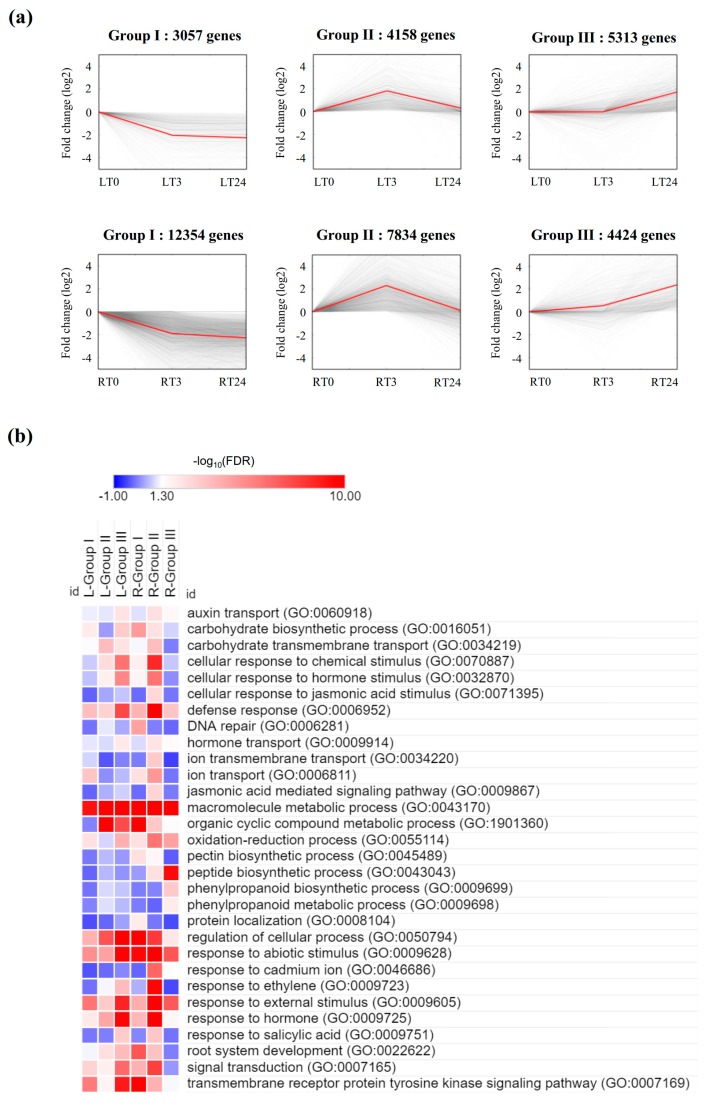
Patterns of gene expressions and Gene ontology (GO) enrichment across three time points in leaves and roots. (**a**) Patterns of gene expressions across three time points in leaves and roots inferred by K-means clustering analysis. In each frame, the light gray lines represented the expression pattern of each gene while the red line represented the expression tendency of all the genes. (**b**) GO enrichment analysis of three significant clusters in leaves and roots. The significance of the most represented GO-slims in each main cluster is indicated by false discovery rate (FDR) value. The red areas represented the significant FDR values, whereas the white and blue represented the nonsignificant values.

To have an overall picture of the impact of Cd stress on transcriptional profiling, DEGs were identified in leaves and roots at different time points and were grouped by K-means clustering. In this part of the analysis, the criteria for considering a gene as differentially expressed was a fold-change of two or more (|log_2_ fold-change| > 1) and *q* < 0.05. Then gene expression maps were created (Figure 4a). Gene expression trends can be produced by converting dynamic and quantitative gene expression time series into the qualitative term “increasing”, “decreasing”, and “constant” in order to denote what happened during a short time interval, which would help discover similar transcriptional behaviors in a short time frame linking to a biological process [17]. In both roots and leaves, most expression trends were clustered into three groups: group I (decreasing-decreasing), group II (increasing-decreasing), and group III (constant-increasing). In group I (decreasing-decreasing), we observed that a scale difference in DEG numbers between roots and leaves. Much more genes in roots decreased at the interval T0 to T3 and decreased later at T3 to T24. It is plausible that enriched pathways in this group were regulations of cellular, organic cyclic compound metabolic, protein localization, DNA repair, root system development, macromolecule metabolic and so on, which indicated that gene expression involved in these essential biological processes were negatively affected by Cd (Figure 4b). Meanwhile, the different concentration of Cd in leaves and roots can definitely play a dose-effect on the scale of the changes of the transcriptional profiles in this group of genes. Genes involved in phenylpropanoid biosynthesis as well as metabolism, and peptide synthesis were only enriched in group III in roots, which indicated that the activation of this pathway was essential in Cd response at an early stage. Genes involved in auxin transport, carbohydrate transmembrane transport, cellular response to chemical stimulus, defense response and hormone signaling transduction pathway were enriched in group II for roots and in group III for leaves. Cellular response to jasmonic acid (JA) and JA-mediated signaling pathway was only enriched in group II in roots (Figure 4), which indicated that the plants were trying to minimize the damage of induced JA response, just as previous reports suggested that heavy metal stress could induced JA and exogenous applied JA could further increase the damaging effect of metal [18]. These results indicated an obvious organ difference in genes and pathways response to Cd stress.

**Figure 5:**
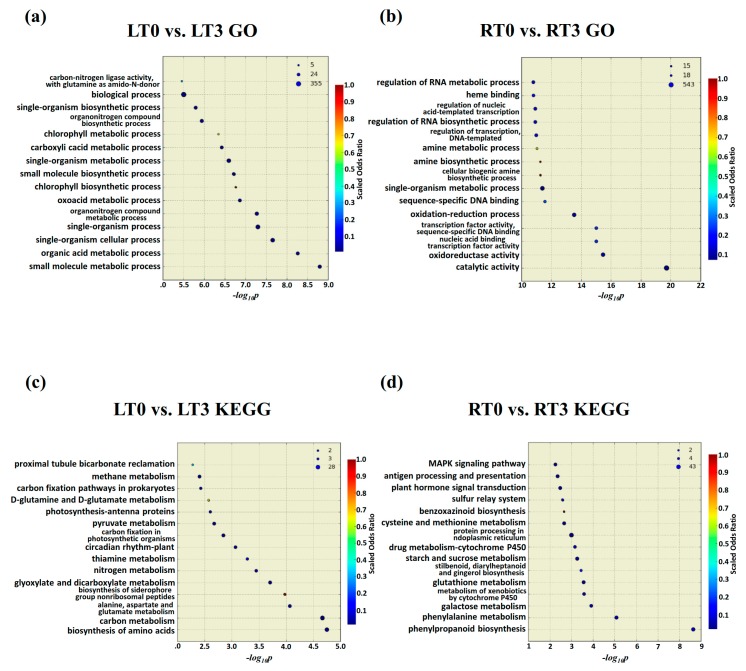
GO and KEGG enrichment analysis of all DEGs following CdCl_2_ exposure at T3 vs. T0 in leaves (**a**,**b**) and roots (**c**,**d**).

Consistent with above analysis, during the first 3 h, GO enrichment and KEGG enrichment analysis demonstrated that much more complicated molecular functions were activated due to the dramatic change of Cd uptake in roots (Figure 5b,d). For example, genes enriched in the pathways such as oxidoreductase activity and transcription binding activities were over-represented during this course by GO analysis (Figure 5b), and several distinct metabolic pathways such as phenylpropanoid biosynthesis, phenylalanie metabolim, galactose metabolism, metabolism of xenobiotics by cytochrome P450, and glutathione metablolism were over-represented by KEGG analysis (Figure 5d). In contrast, DEGs in leaves upon the stress treatment in the first 3 h were enriched in a few general metabolic processes such as biosynthesis of amino acids, biological molecules, and single organism cellular process (Figure 5a,c), which indicated the consistence with the fact that mild but broad damage was caused to these metabolic process due to the slight increase of Cd concentration (Figure 1).

### 2.6. The Induction of Antioxidant Enzyme-Related Genes May Be Closely Associated with Cd Stress Responses

**Figure 6:**
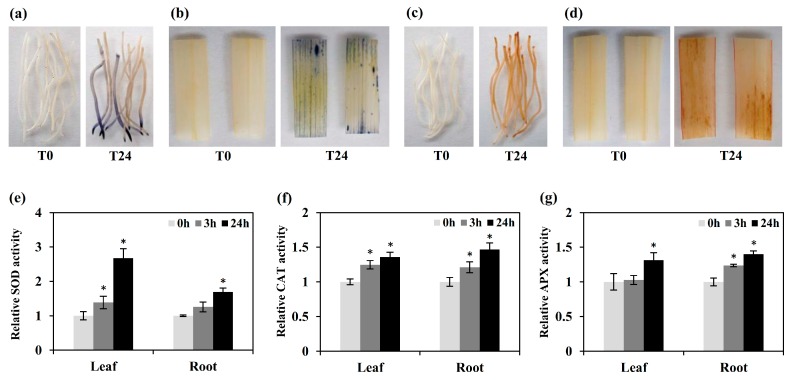
Measurement of ROS levels and anti-oxidative enzymes activity. Detection of O_2_^−^ in leaf (**a**) and root (**b**) of king grass following CdCl_2_ exposure at T0 and T24. Detection of H_2_O_2_ in leaf (**c**) and root (**d**) of king grass following CdCl_2_ exposure at T0 and T24. (**e**) SOD activity. (**f**) CAT activity. (**g**) APX activity. Data are mean ± SD from three replicates. “*” denotes the significant difference between T0 and other time points at *p* < 0.05 by *t*-test.

**Figure 7:**
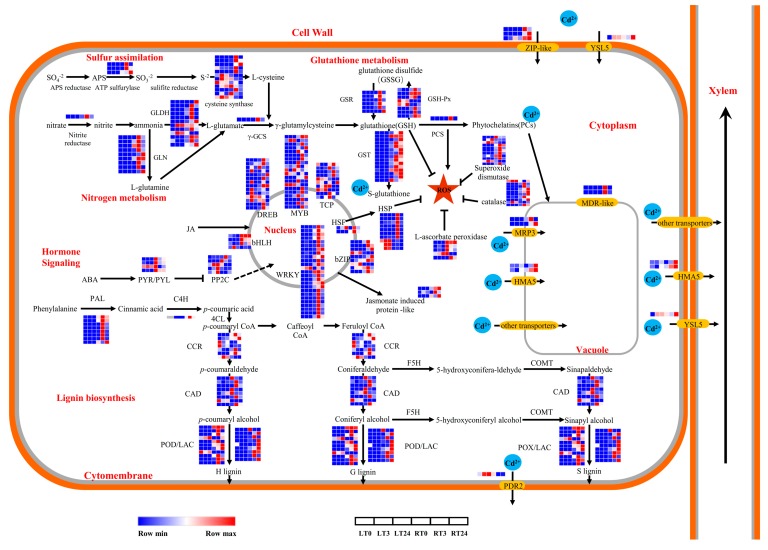
Transcriptional changes of genes responsible for Cd responses in leaves and roots of king grass. The expression heatmaps from left to right were arranged in the following order: leaves—0 h (LT0), leaves—3 h (LT3), leaves—24 h (LT24), roots—0 h (RT0), roots—3 h (RT3) and roots—24 h (RT24).

In response of heavy metal stress, a large amount of reactive oxygen free radicals may be generated and a variety of antioxidant defense systems in plants may be activated to prevent membrane lipid peroxidation, and protect cells from oxidative stress [19,20]. To examine the oxidation-reduction and ROS homeostasis in early king grass Cd response, the generation of ROS was assayed in following CdCl_2_ exposure. Leaves and roots were stained with NBT and DAB for O_2_^−^ and H_2_O_2_, respectively. The ROS level was higher in leaves and roots of king grass treated with Cd at T24 than that at T0 (Figure 6a–d). The superoxide dismutase (SOD), catalase (CAT), and ascorbate peroxidase (APX) activities were enhanced in leaves and roots of king grass at T3 or T24 (Figure 6e–g). Correspondingly, encoding antioxidant enzyme-related genes, such as *CAT*, glutathione reductase (*GSR*), glutathione peroxidase (*GSH-Px*), *APX* and *SOD* were induced at T3 or T24 in king grass (Figure 7). As the first line of defense for the antioxidant defense system, SOD can catalyze the disproportionation of two O_2_^−^ to produce H_2_O_2_ and O_2_. H_2_O_2_ is then rapidly decomposed into H_2_O and O_2_ by POD, GSH-Px, APX and CAT, thus preventing H_2_O_2_ and O_2_^−^ accumulation in the plants [21]. Overexpressing the *Sedum alfredii* *Cu/Zn SOD* gene in transgenic *Arabidopsis* plants enhanced ROS-scavenging capability and exhibited larger Cd uptake capacity in roots under Cd stress [22]. Considering these results together, the induction of antioxidant enzyme-related genes may be closely associated with Cd stress responses in king grass.

Glutathione (GSH) is an important non-enzymatic antioxidant in cells, which protects the sulfhydryl and membrane systems of enzymes and structural proteins, effectively scavenging free radicals produced by Cd stress in plants and resisting peroxidative damage [23]. In our study, ATP sulfurylase and cysteine synthase are key enzymes for cysteine biosynthesis pertaining to a sulfur assimilation pathway related to GSH precursor biosynthesis, which were significantly up-regulated in king grass at both T3 or T24 (Figure 7 and Table S2). Similarly, most of the genes involved in the nitrogen metabolism pathway, such as nitrate reductase, glutamine synthetase (GLN), glutamate dehydrogenase (GLDH) and γ-glutamylcysteine synthetase (γ-GCS) were significantly up-regulated under Cd stress in roots and leaves, consistent with the previous results obtained in pakchoi and water spinach [24,25].

Furthermore, GSH conjugated with Cd^2+^ in cells under the action of glutathione S-transferase (GST) can transport toxic substances into vacuoles [26], thus sequestrating the toxic. GSH can also help to synthesize PCs, kinds of heavy metal complexing peptides for Cd detoxification in plants, catalyzed by phytochelatin synthase (PCS). Our study revealed 15 GST-like encoding genes with high expression levels in king grass at T3 or T24. The induction expression of PCS encoding gene in roots was observed at T3 upon Cd stress (Figure 7 and Table S2). Considering that the increase in Cd accumulation was caused by the augmentation in PC generation proposed by Clemens et al. [27], it indicated that the conjugation of GSH-Cd might play a key role for responding Cd stress in king grass.

### 2.7. Enhanced Cell Wall Biosynthesis Might Play a Vital Role in Cd Stress Tolerance and Cd Ion Accumulations

The cell wall is one of the main components of plant cells, and it is closely related to the differentiation, expansion, and variation of plant cells [28,29]. As the first barrier of metal ion into the cytoplasm, plant root cell wall has a significant fixation effect on heavy metal ions, and is also a site of functional signaling molecules and metabolism in response to heavy metal stress, actively participating in plant response to heavy metal stress process [30,31]. The strengthening of cell wall can be understood as a process of enhancing the barrier in order to hinder the entrance of metal ions. Hence, plants might actively modify its cell wall termed as “cell wall remodeling” which is considered a measure to build more effective barrier in response to metal stress [32]. In *Lupinus albus*, the cell walls fixed twice the amount of Cd ions than the ions bound to the intracellular thiol compounds [33]. As the main component of the secondary wall of plant cells, lignin is a complex polymer formed by many simple phenylpropane and derivatives which has important physiological significance in plant metabolism, for the changes of its enzyme activities, intermediates and lignin content are closely related to plant growth and development, stress tolerance, and other physiological activities [34,35]. Lignification can potentially increase the impenetrability of the cell wall. Moreover, the Cd ions can bind to the cell wall itself [36].

Curiously, our KEGG enrichment analysis showed that phenylpropanoid biosynthesis and phenylalanine metabolism were significantly enriched at both T3 and T24 in roots, and phenylalanine metabolism was enriched in leaves at T24 (Figure 5d and Figure S3d). These results agreed with the discovery in *Arabidopsis* that an early induction of several genes encoding enzymes involved in the biosynthesis of phenylpropanoids were observed [37]. Meanwhile, in this study, most of the genes encoding key enzymes for lignin biosynthesis, including phenylalanine ammonia-lyase (PAL), cinnamate 4-hydroxylase (C4H), cinnamoyl-CoA reductase (CCR), cinnamyl alcohol dehydrogenase (CAD), laccase (LAC) and peroxidase (POD) were induced by Cd treatment in roots and leaves (Figure 7 and Table S2). The above-mentioned results were in agreement with the observations in both woody and herbaceous plants that both the expression of key enzymes involved in lignin biosynthesis and lignification in response to Cd stress were increased [5,38]. In both Cd-sensitive and tolerant varieties of *Vicia sativa*, POD and LAC activities in roots have been increased in response to Cd stress [39], which resulted from increased lignin content. However, the accumulation of lignin content and POD activities were higher in the Cd-tolerant variety comparing to sensitive variety. The data indicated that the aforementioned enzymes in lignification indeed play a prominent role and can carry a strong weight in determining the tolerance ability of plants exposed to Cd stress.

Based on the above analysis, we concluded that the transcriptional changes in cell wall remodeling pathway resulted from early Cd stress response might participate in the process of Cd stress tolerance and Cd ion accumulations in king grass.

### 2.8. Transporter Activities Were Activated in Early Cd Response

Cd is one of the non-essential heavy metal elements of plants. It is mainly introduced into plants through the absorption pathway of divalent cations such as manganum (Mn^2+^), iron (Fe^2+^), calcium (Ca^2+^) and zinc (Zn^2+^) via roots [40]. For example, ZIP (Zinc regulated transporter/iron-regulated transporter-like protein) transporter participates on the accumulation of metal ions, including Zn^2+^, Fe^2+^ and Mn^2+^. As well as this, it also works on plant heavy metal detoxification [41]. In more detail, one of the most important players in Cd transport, especially for the entry of Cd into plants, is Zinc/Fe transporter IRT1 [42]. Overexpression of *IRT1* could result in increased Cd accumulation in roots of *Arabidopsis* and rice [43,44]. It has been found that *IRT1* was also involved in the high-affinity Cd uptake by roots in a species to hyper-accumulate Cd, *Thlaspi caerulescens* L. [45]. According to recent reports on ryegrass plants, many genes of IRT family (*IRT4*, *IRT6*, *IRT7*, *IRT8*, and *IRT10*) were significantly up-regulated under Cd stress [46]. Further, increased expression of *IRT* genes in roots and stems could lead to enhanced tolerance to Cd stress, and increased expression of *IRT* in leaves could enhance the transfer of Cd from roots to leaves in ryegrass [46]. In our study, another transporter family ZIPs were found to be DEGs, among which *TRINITY_DN274974_c0_g1_i1* was induced by Cd stress in both leaves and roots, and had higher expression in roots than in leaves at all three time points, while *TRINITY_DN150429_c0_g1_i1* and *TRINITY_DN274667_c1_g2_i2* were induced by Cd stress only in roots (Figure 7; Table S2). These results indicated that different ZIP transporters might be involved in early Cd stress response.

The ATP-binding cassette (ABC) transporter family is one of the largest families of membrane proteins, including pleiotropic drug resistance protein (PDR), multidrug resistance protein (MDR) and multidrug resistance-associated protein (MRP) [47]. These three types of ABC transporters can transport a wide range of heavy metals such as arsenic (As), Cd, stibium (Sb), mercury (Hg) and Zn, participating in trans-tonoplast transport of bis(glutathionato)cadmium (GS_2_-Cd) and Cd-bound phytochelatin (PC-Cd) complexes as Cd pumps. Some ABC transporters also have the function of transporting GS_2_-Cd and PC-Cd complexes into subcellular compartments or outside of the cell [48,49]. For example, PDR8, as a member of the PDR subfamily of ABC transporters in *Arabidopsis*, majorly appears in the root epidermal cell plasma membranes, pumps Cd^2+^ out of the cytosol, and transports them to the apoplast to decrease the Cd concentration [50]. Moreover, it was found in Chinese cabbage that the expression of *PDR8* gene in low-Cd-enriched genotype (LAKJ) was much higher than that in highly Cd enriched genotype (HAJS), which reduced the adsorption of Cd in LAKJ, thereby reducing the enrichment in leaves [25]. Wojas et al. [51] found that the heterologous expression of *AtMRP7* in tobacco is localized in the tonoplast and plasma membrane, which can transport Cd into the vacuoles of the leaves, increasing the Cd content in the vacuoles, thereby reducing the toxicity of Cd to tobacco. Additionally, overexpressing *YCF1*, a yeast MRP homolog gene, could transport Cd-GS_2_ complexes into the vacuoles, which showed greater accumulation of Cd in *Arabidopsis* [52]. In this study, five *ABC transporter* encoding genes (MRP-like: *TRINITY_DN255923_c2_g1_i1*, *TRINITY_DN393756_c0_g1_i1*; MDR-like: *TRINITY_DN181964_c0_g1_i1*, *TRINITY_DN238545_c0_g1_i1*; PDR-like: *TRINITY_DN264698_c0_g1_i1*) were identified as DEGs (Figure 7 and Table S2). Interestingly, the former four were significantly up-regulated under Cd stress only in roots of king grass. We concluded that these genes may play crucial roles in Cd uptake in king grass. Further study of the precise molecular function may benefit for revealing the mechanisms of differential Cd accumulation in roots and shoots of king grass.

After Cd ions are absorbed into the plants through the roots, they are usually separated into vacuoles [53]. In rice, this process is carried out by the P_1B_-type ATPase (heavy metal transporting ATPase, HMA) protein OsHMA3 [12]. In the T-DNA insertional mutant *oshma3* or the loss-of-function allele of *OsHMA3* cultivars, Cd is not transported into the vacuole, resulting in a significant increase in the transport from roots to shoots and grains [54]. In contrast, overexpression of the *OsHMA3* gene can trap Cd in the roots, significantly reducing Cd accumulation in the grains [55]. In *Arabidopsis*, there are eight members of HMA family proteins [56], in which AtHMA3 is closely related to vacuolar sequestration of Zn, Cd, cobalt (Co) and plumbum (Pb) [57], as well as AtHMA2 and AtHMA4 play key roles in xylem loading of Cd and Zn [58,59]. More recently, studies in sweet sorghum have shown that a homologous HMA family gene *Sb04g006600* plays a key role in the response to Cd stress, and it is speculated that the difference in Cd resistance between different varieties is not caused by differences in expression changes, which may be determined by differences in function [60]. In our studies, two HMA family genes *TRINITY_DN202963_c0_g1_i1* and *TRINITY_DN218483_c1_g1_i1* were up-regulated in response to Cd stress at T3 and T24 (Figure 7; Table S2). Therefore, in-depth study of the detailed molecular mechanism of different HMA family gene members in response to Cd stress in king grass is of great significance for the subsequent generation of high and low Cd-enriched king grass varieties by means of in situ overexpression or CRISPR/Cas9 gene editing technology gene knockout HMA gene.

Besides from the form of Cd^2+^, Cd can also enter root cells through the forms of Cd-chelates by YSL (yellow stripe 1-like) proteins [42], which also participated the transport of Cd from symplasm into xylem [61]. In our study, YSL5-like encoding gene (*TRINITY_DN290298_c0_g1_i2*) was identified to be DEGs (Figure 7 and Table S2), which was significantly up-regulated under Cd stress in roots and leaves at T3 and T24 suggesting that YSL5-like might participate in Cd transport. However, the exact role it played in Cd stress response in king grass remains to be further investigated.

### 2.9. Hormone Signal Transduction Pathway Responded in King Grass

The first barriers of plant Cd stress defense are cell walls and cell membranes including the induction of membrane-localized NADPH oxidase and accumulation of signaling molecules such as calcium ions and nitric oxide (NO) [62]. The calcium signals can then induce other signaling molecules such as jasmonic acid (JA), salicylic acid (SA), or abscisic acid (ABA) [63]. These plant hormone signaling molecules can be transmitted through their induced protein, thus impacting the gene regulation by modulating the expression of transcription factors (TFs) including MYB, WRKY, or DREB families or other defense proteins [64]. In this study, we observed that at least 3 predicted JA-induced protein (*TRINITY_DN295832_c3_g6_i3*, *TRINITY_DN280901_c0_g1_i2*, *TRINITY_DN248031_c0_g1_i1*) were significantly up-regulated upon Cd stress in king grass roots but not in leaves (Figure 7 and Table S2). The member PYR/PYL (pyrabactin resistant) and PP2Cs (A-group proteins phosphatase; 2C) of ABA signal transduction pathway were activated in king grass (Figure 7). Moreover, different TFs families, including MYB, WRKY, HSF, bHLH, bZIP, DREB, HSF and TCP, were identified as significantly induced or repressed in king grass roots and leaves by Cd stress (Figure 7 and Table S2). Elevated JA levels were observed in several plant species such as runner bean [65], and *Arabidopsis* [66]. It has been indicated that JA has protective effects at lower concentration which could improve plant antioxidant response system by promoting the activities classic antioxidant enzymes CAT and SOD or enhancing the GSH content [64]. However, the JA level at higher concentration (10^−4^ mol/L) could negatively impact plant growth such as growth reduction, chlorophyll degradation, and photosynthesis decrease [67]. Combining with our gene expression data on GSH and antioxidant enzymes, JA-induced protein in the first 24 h in roots may play a positive role in promoting these activities. Furthermore, detailed studies on JA or JA-induced protein need to be carried out to explore the involvement of these important signaling molecules.

In conclusion, significantly high Cd uptake in roots in the first 3 h might be attributed to faster activation of transcriptomic responses. Significant but slight increase of Cd content in leaves in the first 24 h was consistent with our GO enrichment analysis result that ion transport network was enriched. Detoxification process such as oxidation-reduction played an important role in the first 3 h for roots and in the first 24 h for leaves. Transporter activity and JA signaling pathway were activated to some extent and may be connected to GSH activity.

## 3. Materials and Methods

### 3.1. Plant Growth and Cd Treatments

The king grass (*Pennisetum americanum* × *P. purpureum*) cultivar “Bangde NO.1” was provided by Shi ji tian yuan Co. (Henan, China). The seeds were sterilized in 20% chlorine bleach for 5 min and rinsed four times with deionized water. Fifty seeds per pot (30 cm length, 16 cm width, 10 cm deep) were sown and irrigated with 1/2 strength Hoagland’s solution in growth chambers. The plants were grown in the temperature of 25 °C in the daytime and 20 °C in the night with photosynthetically active radiation 400 μmol m^−2^·s^−1^. After two months of growth, the plants were treated with 100 µM cadmium (CdCl_2_) solution. Cd-treated and untreated leaf and root samples were collected at T0, T3 and T24 time points after applying the Cd treatments. All the sample tissues were treated with liquid nitrogen first and immediately stored in the −80 °C freezer for further analysis. Three replicates for each organ type and time point were harvested and subjected to RNA-seq.

### 3.2. Cd Concentration Measurements in Leaves and Roots

To detect the Cd concentration in roots and leaves of king grass, three replicates per each organ type and time point were dried in 80 °C and then digested by HNO_3_ and H_2_O_2_ mixture solution in a microwave digester. ICP Mass Spectrometer (NexION 300D, Waltham, MA, USA) was used to measure the Cd concentration with the manufacturer’s instruction. Statistical significance analysis on samples in response to different Cd concentrations at each time point was conducted using One-way Analysis of Variance (ANOVA) and the least significant difference (LSD) tests performed with SPSS13.0.

### 3.3. Determination of ROS Accumulation and the Activities of Antioxidant Enzyme

O_2_^−^ and H_2_O_2_ were stained according to the methods of Dutilleul [68]. The activities of CAT and APX were determined according to the method of Chance and Maehly [69]. The activity of SOD was determined using a Total SOD Assay Kit (S0102; Haimen Beyotime, Haimen, China).

### 3.4. RNA Extraction, Sequencing, and De Novo Assembly

#### 3.4.1. RNA Extraction

The RNeasy Plant Mini Kit (Qiagen, Germantown, MD, USA) was used to extract and purify the total RNA of roots and leaves from all samples following the manufacture’s manual.

#### 3.4.2. Library Construction

(1) mRNA enrichment and purification: Poly (T) oligo-attached magnetic beads were used to purify and enrich the Poly (A)-containing mRNA molecules, and then the enriched mRNAs were subjected to fragmentation. (2) First-strand cDNA synthesis: Using random hexamers, first-strand cDNAs were reverse transcribed. (3) Second-strand cDNA synthesis: dUTP was used to synthesize the second-strand cDNA. These generated cDNAs were then subjected to purification. (4) End polishing, A-tailing, and adaptor ligation: End repair and 3′ adenylation were performed to treat newly synthesized cDNAs generated by the above steps. The 3′ adenylated cDNA fragments were ligated to adaptors. (5) Second-strand cDNA degradation: UDG (Uracil—DNA Glycocasylase) was used to degrade second-strand cDNAs. (6) PCR amplication: The ligation products were purified from TAE gel, subjected to PCR amplication. (7) Library quality control: Library was validated on the Agilent Technologies 2100 Bio-analyzer and the Bio-Rad CFX96 Real-Time PCR System. (8) Sequencing: The Illumina HiSeq 2000 platform was used to conduct sequencing of each library.

#### 3.4.3. De Novo Assembly

The raw sequence data was processed to remove poor quality sequences and trim the adaptor sequences by using cutadapt (v1.8.1). The clean sequencing data for each sample were assembled to transcripts by the utilization of trinity software [70]. Cd-hit (v4.6) was used to cluster all assembled transcripts from total samples [71]. The alignment to Rfam database (v.11) was followed to compare the clustered transcripts (http://www.sanger.ac.uk/Software/Rfm/) to get rid of the matching ribosomal RNAs. The software transdecoder (v.2.0.1) was used to predict the open reading frames of the transcripts.

### 3.5. Normalization of Gene Expression and DEGs Identification

The sequencing reads were used to map the remaining transcripts without rRNAs. To quantify total expressed transcripts, the RSEM method with the TPM (Transcripts per million) value was adopted [72]. Differential expressed genes were identified using the R language (v3.2.1) with edgeR package [73]. The fold change between the two groups was calculated as: logFC = log_2_ (One time point/Another time point) or logFC = log_2_ (Root/Leaf). Genes in two groups, whose |logFC| > 1 and *q* value < 0.05, were defined as differential expressed genes in this study.

### 3.6. Gene Expression Trend Analysis

Gene groupings were performed by K-means clustering. Gene expression level was scaled across samples and the line plot was created by Python Matplotlib. The heatmap was created with morpheus (https://software.broadinstitute.org/morpheus/).

### 3.7. Functional Annotation

The expressed transcripts’ function was annotated using the ‘blastp’ program (v. 2.2.29) and NR, Swissport, and eggNOG database with the cutoff of e-value < 1e-5. The Gene Ontology (GO) terms, the gene families, and the domains were annotated and assigned by the software interproscan (v.5.15-54.0). KEGG pathways were mapped by using KEGG Annotation.

### 3.8. Gene Expression Validation by qRT-PCR

The first-strand cDNA was synthesized using PrimeScript™ RT reagent Kit (TaKaRa, Beijing, China) according to the manufacturer’s protocol. Quantity real-time PCR was conducted by using Sosofast EvaGreen Supermix kit (Bio-Rad, Hercules, CA, USA) and Bio-Rad CFX96 real-time PCR detection system. Each 20 μL q-PCR reaction includes: 1 μL forward and reverse primers with the final primer concentration 0.2 μM, 10 μL qPCR EvaGreen Supermix, 2μL cDNA, and 7 μL ddH_2_O. The real-time PCR running protocol was as following: pre-denaturation for 10 s in 94 °C, denaturation for 10 s in 94 °C, annealing 10 s in 60 °C, repeat for 40 cycles. Three biological replicates and three technical replicates were included for each sample. *PgEIF4A* was used as the reference to quantify the gene expression level in king grass [74]. All primers used in our qRT-PCR analysis were listed in Table S1.

## Supplementary Materials

Supplementary materials can be found at https://www.mdpi.com/1422-0067/20/10/2532/s1. Figure S1: Correlation between qRT-PCR and RNA sequencing for the 15 selected genes. Data was log10 transformed. Figure S2: Expression of the selected 15 genes inferred at T0, T3 and T24 by RNA-seq and qRT-PCR. L and R represents leaves and roots, respectively. Error bars represent standard deviation (*n* = 3). Figure S3: GO and KEGG enrichment analysis of all DEGs following CdCl_2_ exposure at T24 vs. T0 and T24 vs. T3 in leaves. Figure S4: GO and KEGG enrichment analysis of all DEGs following CdCl_2_ exposure at T24 vs. T0 and T24 vs. T3 in roots. Table S1: The primers used in qRT-PCR analysis. Table S2: List of differentially expressed genes (DEGs).

## References

[1] Tchounwou P.B., Yedjou C.G., Patlolla A.K., Sutton D.J., Luch A. (2012). Heavy metal toxicity and the environment. Molecular, Clinical and Environmental Toxicology.

[2] Rizwan M., Ali S., Abbas T., Zia-Ur-Rehman M., Hannan F., Keller C., Al-Wabel M.I., Yong S.O. (2016). Cadmium minimization in wheat: A critical review. Ecotoxicol. Environ. Saf..

[3] Rizwan M., Ali S., Adrees M., Ibrahim M., Tsang D.C.W., Zia-Ur-Rehman M., Zahir Z.A., Rinklebe J., Tack F.M.G., Yong S.O. (2017). A critical review on effects, tolerance mechanisms and management of cadmium in vegetables. Chemosphere.

[4] Ali H., Khan E., Sajad M.A. (2013). Phytoremediation of heavy metals-concepts and applications. Chemosphere.

[5] Yang J., Li K., Zheng W., Zhang H., Cao X., Lan Y., Yang C., Li C. (2015). Characterization of early transcriptional responses to cadmium in the root and leaf of Cd-resistant *Salix matsudana* Koidz. BMC Genom..

[6] Zhang X.F., Zhang X.H., Gao B., Li Z.A., Xia H.P., Li H.F., Li J. (2014). Effect of cadmium on growth, photosynthesis, mineral nutrition and metal accumulation of an energy crop, king grass (*Pennisetum americanum* × *P. purpureum*). Biomass Bioenerg.

[7] Fan X.F., Hou X.C., Zhu Y., Wu J.Y. (2012). Biomass yield and quality of hybrid pennisetum. Chin. J. Grassl..

[8] Wang J., Xin D., Hou X., Wu J., Fan X., Li K., Zhang J. (2016). Structural properties and hydrolysabilities of chinese pennisetum and hybrid pennisetum: Effect of aqueous ammonia pretreatment. Bioresour. Technol..

[9] Mendoza-Cozatl D.G., Butko E., Springer F., Torpey J.W., Komives E.A., Kehr J., Schroeder J.I. (2008). Identification of high levels of phytochelatins, glutathione and cadmium in the phloem sap of *Brassica napus.* A role for thiol-peptides in the long-distance transport of cadmium and the effect of cadmium on iron translocation. Plant J..

[10] Guo W.J., Meetam M., Goldsbrough P.B. (2008). Examining the specific contributions of individual *Arabidopsis* metallothioneins to copper distribution and metal tolerance. Plant Physiol..

[11] Grennan A.K. (2009). Identification of genes involved in metal transport in plants. Plant Physiol..

[12] Ueno D., Iwashita T., Zhao F.J., Ma J.F. (2008). Characterization of Cd translocation and identification of the Cd form in xylem sap of the Cd-hyperaccumulator *Arabidopsis halleri*. Plant Cell Physiol..

[13] Lee K., Bae D.W., Kim S.H., Han H.J., Liu X., Park H.C., Lim C.O., Lee S.Y., Chung W.S. (2010). Comparative proteomic analysis of the short-term responses of rice roots and leaves to cadmium. J. Plant Physiol..

[14] He J., Li H., Luo J., Ma C., Li S., Qu L., Gai Y., Jiang X., Janz D., Polle A. (2013). A transcriptomic network underlies microstructural and physiological responses to cadmium in *Populus* × *canescens*. Plant Physiol..

[15] Jozefczak M., Keunen E., Schat H., Bliek M., Hernandez L.E., Carleer R., Remans T., Bohler S., Vangronsveld J., Cuypers A. (2014). Differential response of *Arabidopsis* leaves and roots to cadmium: Glutathione-related chelating capacity vs. antioxidant capacity. Plant Physiol. Biochem..

[16] Shamshad S., Shahid M., Rafiq M., Khalid S., Dumat C., Sabir M., Murtaza B., Farooq A.B.U., Shah N.S. (2018). Effect of organic amendments on cadmium stress to pea: A multivariate comparison of germinating vs. young seedlings and younger vs. older leaves. Ecotoxicol. Environ. Saf..

[17] Wabnik K., Hvidsten T.R., Kedzierska A., Van Leene J., De Jaeger G., Beemster G.T.S., Komorowski J., Kuiper M.T.R. (2009). Gene expression trends and protein features effectively complement each other in gene function prediction. Bioinformatics.

[18] Per T.S., Khan M.I.R., Anjum N.A., Masood A., Hussain S.J., Khan N.A. (2018). Jasmonates in plants under abiotic stresses: Crosstalk with other phytohormones matters. Environ. Exp. Bot..

[19] Hsu Y.T., Kao C.H. (2007). Toxicity in leaves of rice exposed to cadmium is due to hydrogen peroxide accumulation. Plant Soil.

[20] Shah K., Kumar R.G., Verma S., Dubey R.S. (2001). Effect of cadmium on lipid peroxidation, superoxide anion generation and activities of antioxidant enzymes in growing rice seedlings. Plant Sci..

[21] Baxter A., Mittler R., Suzuki N. (2014). ROS as key players in plant stress signalling. J. Exp. Bot..

[22] Li Z., Han X., Song X., Zhang Y., Jiang J., Han Q., Liu M., Qiao G., Zhuo R. (2017). Overexpressing the *Sedum alfredii* Cu/Zn superoxide dismutase increased resistance to oxidative stress in transgenic *Arabidopsis*. Front. Plant Sci..

[23] Alscher R.G. (2010). Biosynthesis and antioxidant function of glutathione in plants. Physiol. Plant..

[24] Huang Y.Y., Shen C., Chen J.X., He C.T., Zhou Q., Tan X., Yuan J.G., Yang Z.Y. (2016). Comparative transcriptome analysis of two *Ipomoea aquatica* Forsk. Cultivars targeted to explore possible mechanism of genotype-dependent accumulation of cadmium. J. Agric. Food Chem..

[25] Zhou Q., Guo J.J., He C.T., Shen C., Huang Y.Y., Chen J.X., Guo J.H., Yuan J.G., Yang Z.Y. (2016). Comparative transcriptome analysis between low- and high-cadmium-accumulating genotypes of pakchoi (*Brassica chinensis* L.) in response to cadmium stress. Environ. Sci. Technol..

[26] Marrs K.A. (1996). The functions and regulation of glutathione S-transferases in plants. Annu. Rev. Plant Physiol. Plant Mol. Biol..

[27] Clemens S., Kim E.J., Neumann D., Schroeder J.I. (1999). Tolerance to toxic metals by a gene family of phytochelatin synthases from plants and yeast. EMBO J..

[28] Hueckelhoven R. (2007). Cell wall—Associated mechanisms of disease resistance and susceptibility. Ann. Rev. Phytopathol..

[29] Roberts K. (2001). How the cell wall acquired a cellular context. Plant Physiol..

[30] Chen G., Liu Y., Wang R., Zhang J., Owens G. (2013). Cadmium adsorption by willow root: The role of cell walls and their subfractions. Environ. Sci. Pollut. Res..

[31] DalCorso G., Farinati S., Furini A. (2010). Regulatory networks of cadmium stress in plants. Plant Signal. Behav..

[32] Loix C., Huybrechts M., Vangronsveld J., Gielen M., Keunen E., Cuypers A. (2018). Reciprocal interactions between cadmium-induced cell wall responses and oxidative stress in plants. Front. Plant Sci..

[33] Vázquez S., Goldsbrough P., Carpena R.O. (2010). Assessing the relative contributions of phytochelatins and the cell wall to cadmium resistance in white lupin. Physiol. Plant..

[34] Ithal N., Recknor J., Nettleton D., Maier T., Baum T.J., Mitchum M.G. (2007). Developmental transcript profiling of Cyst nematode feeding cells in soybean roots. Mol. Plant-Microbe Interact..

[35] Magalhaes Silva Moura J.C., Valencise Bonine C.A., Fernandes Viana J.d.O., Dornelas M.C., Mazzafera P. (2010). Abiotic and biotic stresses and changes in the lignin content and composition in plants. J. Integr. Plant Biol..

[36] Parrotta L., Guerriero G., Sergeant K., Cal G., Hausman J.-F. (2015). Target or barrier? The cell wall of early- and later-diverging plants vs. cadmium toxicity: Differences in the response mechanisms. Front. Plant Sci..

[37] Herbette S., Taconnat L., Hugouvieux V., Piette L., Magniette M.L.M., Cuine S., Auroy P., Richaud P., Forestier C., Bourguignon J. (2006). Genome-wide transcriptome profiling of the early cadmium response of *Arabidopsis* roots and shoots. Biochimie.

[38] Kovacik J., Backor M., Kadukova J. (2008). Physiological responses of matricaria chamomilla to cadmium and copper excess. Environ. Toxicol..

[39] Rui H., Chen C., Zhang X., Shen Z., Zhang F. (2016). Cd-induced oxidative stress and lignification in the roots of two *Vicia sativa* L. varieties with different Cd tolerances. J. Hazard. Mater..

[40] Roth U., von Roepenack-Lahaye E., Clemens S. (2006). Proteome changes in *Arabidopsis thaliana* roots upon exposure to Cd^2+^. J. Exp. Bot..

[41] Guerinot M.L. (2000). The ZIP family of metal transporters. Biochim. Biophys. Acta.

[42] Vert G., Grotz N., Dedaldechamp F., Gaymard F., Guerinot M.L., Briat J.-F., Curie C. (2002). IRT1, an *Arabidopsis transporter* essential for iron uptake from the soil and for plant growth. Plant Cell.

[43] Connolly E.L., Fett J.P., Guerinot M.L. (2002). Expression of the IRT1 metal transporter is controlled by metals at the levels of transcript and protein accumulation. Plant Cell.

[44] Lee S., An G. (2009). Over-expression of *OsIRT1* leads to increased iron and zinc accumulations in rice. Plant Cell Environ..

[45] Lombi E., Tearall K.L., Howarth J.R., Zhao F.-J., Hawkesford M.J., McGrath S.P. (2002). Influence of iron status on cadmium and zinc uptake by different ecotypes of the hyperaccumulator *Thlaspi caerulescens*. Plant Physiol..

[46] Chi S., Qin Y., Xu W., Chai Y., Feng D., Li Y., Li T., Yang M., He Z. (2018). Differences of Cd uptake and expression of *OAS* and *IRT* genes in two varieties of ryegrasses. Environ. Sci. Pollut. Res. Int..

[47] Klein I., Sarkadi B., Váradi A. (2000). An inventory of the human ABC proteins. Biochim. Biophys. Acta.

[48] Martinoia E., Klein M., Geisler M., Bovet L., Forestier C., Kolukisaoglu U., Muller-Rober B., Schulz B. (2002). Multifunctionality of plant ABC transporters--more than just detoxifiers. Planta.

[49] Ortiz D.F., Ruscitti T., McCue K.F., Ow D.W. (1995). Transport of metal-binding peptides by HMT1, a fission yeast ABC-type vacuolar membrane protein. J. Biol. Chem..

[50] Kim D.Y., Bovet L., Maeshima M., Martinoia E., Lee Y. (2007). The ABC transporter AtPDR8 is a cadmium extrusion pump conferring heavy metal resistance. Plant J..

[51] Wojas S., Hennig J., Plaza S., Geisler M., Siemianowski O., Sklodowska A., Ruszczynska A., Bulska E., Antosiewicz D.M. (2009). Ectopic expression of *Arabidopsis* ABC transporter MRP7 modifies cadmium root-to-shoot transport and accumulation. Environ. Pollut..

[52] Lu Y.P., Li Z.S., Rea P.A. (1997). *AtMRP1* gene of *Arabidopsis* encodes a glutathione S-conjugate pump: Isolation and functional definition of a plant ATP-binding cassette transporter gene. Proc. Natl. Acad. Sci. USA.

[53] Migocka M., Papierniak A., Kosatka E., Kłobus G. (2011). Comparative study of the active cadmium efflux systems operating at the plasma membrane and tonoplast of cucumber root cells. J. Exp. Bot..

[54] Yan J., Wang P., Wang P., Yang M., Lian X., Tang Z., Huang C.-F., Salt D.E., Zhao F.J. (2016). A loss-of-function allele of *OsHMA3* associated with high cadmium accumulation in shoots and grain of japonica rice cultivars. Plant Cell Environ..

[55] Ueno D., Yamaji N., Kono I., Huang C.F., Ando T., Yano M., Ma J.F. (2010). Gene limiting cadmium accumulation in rice. Proc. Natl. Acad. Sci. USA.

[56] Baxter I., Tchieu J., Sussman M.R., Boutry M., Palmgren M.G., Gribskov M., Harper J.F., Axelsen K.B. (2003). Genomic comparison of P-type ATPase ion pumps in *Arabidopsis* and rice. Plant Physiol..

[57] Williams L.E., Mills R.F. (2005). P(1b)-ATPases--an ancient family of transition metal pumps with diverse functions in plants. Trends Plant Sci..

[58] Hussain D., Haydon M.J., Wang Y., Wong E., Sherson S.M., Young J., Camakaris J., Harper J.F., Cobbett C.S. (2004). P-type ATPase heavy metal transporters with roles in essential zinc homeostasis in *Arabidopsis*. Plant Cell.

[59] Mills R.F., Valdes B., Duke M., Peaston K.A., Lahner B., Salt D.E., Williams L.E. (2010). Functional significance of AtHMA4 C-terminal domain in planta. PLoS ONE.

[60] Feng J.J., Jia W.T., Lv S.L., Bao H., Miao F.F., Zhang X., Wang J.H., Li J.H., Li D.S., Zhu C. (2018). Comparative transcriptome combined with morpho-physiological analyses revealed key factors for differential cadmium accumulation in two contrasting sweet sorghum genotypes. Plant Biotechnol. J..

[61] Verbruggen N., Hermans C., Schat H. (2009). Molecular mechanisms of metal hyperaccumulation in plants. New Phytol..

[62] Chmielowska-Bak J., Gzyl J., Rucińska-Sobkowiak R., Arasimowicz-Jelonek M., Deckert J. (2014). The new insights into cadmium sensing. Front. Plant Sci..

[63] Ku Y.S., Sintaha M., Cheung M.Y., Lam H.M. (2014). Plant hormone signaling crosstalks between biotic and abiotic stress responses. Int. J. Mol. Sci..

[64] Noriega G., Santa Cruz D., Batlle A., Tomaro M., Balestrasse K. (2012). Heme oxygenase is involved in the protection exerted by jasmonic acid against cadmium stress in soybean roots. J. Plant Growth Regul..

[65] Rodriguez-Serrano M., Romero-Puertas M.C., Zabalza A., Corpas F.J., Gomez M., Del Rio L.A., Sandalio L.M. (2006). Cadmium effect on oxidative metabolism of pea (*Pisum sativum* L.) roots. Imaging of reactive oxygen species and nitric oxide accumulation in vivo. Plant Cell Environ..

[66] Maksymiec W., Wianowska D., Dawidowicz A.L., Radkiewicz S., Mardarowicz M., Krupa Z. (2005). The level of jasmonic acid in *Arabidopsis thaliana* and *Phaseolus coccineus* plants under heavy metal stress. J. Plant Physiol..

[67] Maksymiec W., Krupa Z. (2002). Jasmonic acid and heavy metals in *Arabidopsis* plants—A similar physiological response to both stressors?. J. Plant Physiol..

[68] Dutilleul C., Garmier M., Noctor G., Mathieu C., Chetrit P., Foyer C.H., Paepe R.D. (2003). Leaf mitochondria modulate whole cell redox homeostasis, set antioxidant capacity, and determine stress resistance through altered signaling and diurnal regulation. Plant Cell.

[69] Chance B., Maehly A.C. (1955). Assay of catalases and peroxidases. Methods Enzymol..

[70] Haas B.J., Papanicolaou A., Yassour M., Grabherr M., Blood P.D., Bowden J., Couger M.B., Eccles D., Li B., Lieber M. (2013). *De novo* transcript sequence reconstruction from RNA-seq using the trinity platform for reference generation and analysis. Nature Protoc..

[71] Li W.Z., Godzik A. (2006). Cd-hit: A fast program for clustering and comparing large sets of protein or nucleotide sequences. Bioinformatics.

[72] Li B., Dewey C.N. (2011). Rsem: Accurate transcript quantification from RNA-seq data with or without a reference genome. BMC Bioinformatics.

[73] Nikolayeva O., Robinson M.D. (2014). Edger for differential RNA-seq and CHIP-seq analysis: An application to stem cell biology. Methods Mol. Biol..

[74] Reddy P.S., Reddy D.S., Sharma K.K., Bhatnagar-Mathur P., Vadez V. (2015). Cloning and validation of reference genes for normalization of gene expression studies in pearl millet [*Pennisetum glaucum* (L.) R. Br.] by quantitative real-time PCR. Plant Gene.

