# Supplementary material for: Transcriptome Analysis to Shed Light on the Molecular Mechanisms of Early Responses to Cadmium in Roots and Leaves of King Grass (Pennisetum americanum × P. purpureum)
Source: Int J Mol Sci. 2019 May 23;20(10):2532. doi: 10.3390/ijms20102532 (PMC6567004; doi:10.3390/ijms20102532)
Supplement: Supplementary file 1 [file ijms-20-02532-s001.zip › ijms-501562/Manuscript/Supplementary Figures.pptx]

## Slide 1
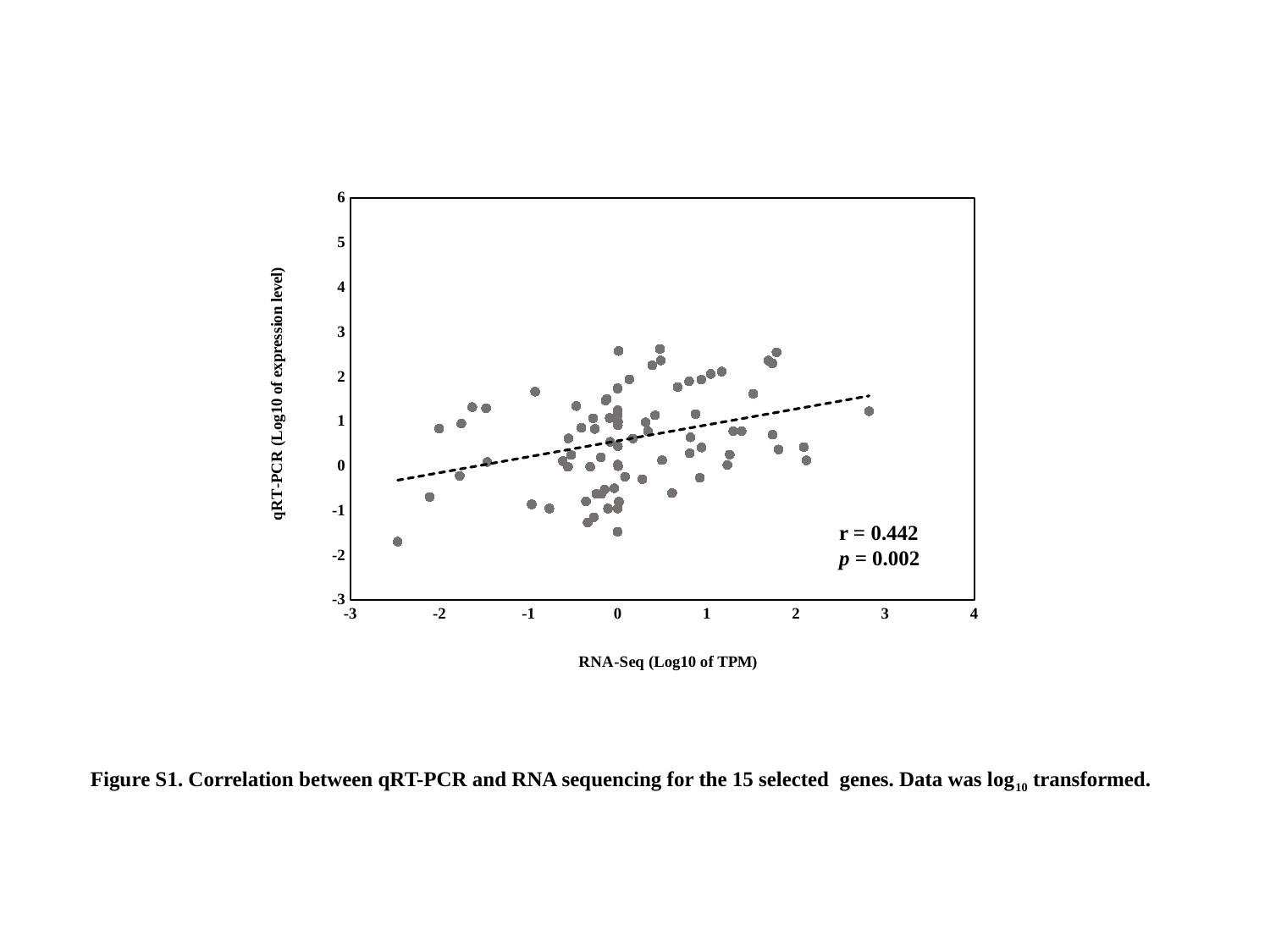

### Chart
| Category | |
|---|---|r = 0.442
p = 0.002
Figure S1. Correlation between qRT-PCR and RNA sequencing for the 15 selected genes. Data was log10 transformed.

## Slide 2
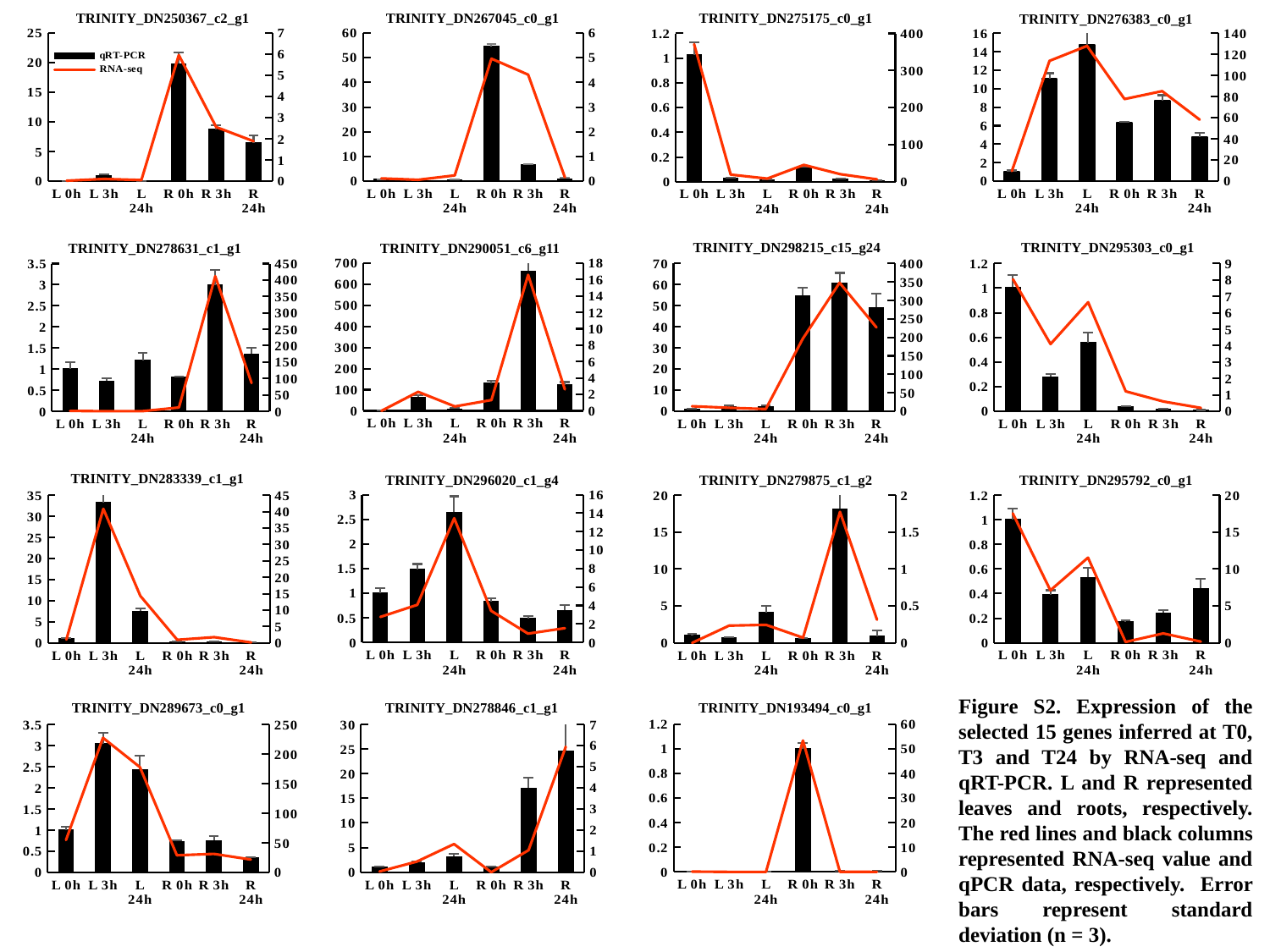

TRINITY_DN250367_c2_g1
TRINITY_DN267045_c0_g1
TRINITY_DN275175_c0_g1
TRINITY_DN276383_c0_g1
### Chart
| Category | qRT-PCR | RNA-seq |
|---|---|---|
| L 0h | 0.0 | 0.02666666666666667 |
| L 3h | 1.0050550154406774 | 0.11 |
| L 24h | 0.0 | 0.056666666666666664 |
| R 0h | 19.814202057554112 | 5.95 |
| R 3h | 8.766181800386107 | 2.5566666666666666 |
| R 24h | 6.464825389228783 | 1.89 |
### Chart
| Category | qRT-PCR | RNA-seq |
|---|---|---|
| L 0h | 0.7806811968936285 | 0.11 |
| L 3h | 0.46111477324379024 | 0.05333333333333334 |
| L 24h | 0.5775983509645909 | 0.2333333333333333 |
| R 0h | 54.70041323344632 | 4.96 |
| R 3h | 6.565575857360353 | 4.3133333333333335 |
| R 24h | 1.0346973362232168 | 0.15666666666666665 |
### Chart
| Category | qRT-PCR | RNA-seq |
|---|---|---|
| L 0h | 1.0102814658576136 | 9.513333333333334 |
| L 3h | 11.09159279747955 | 113.86666666666667 |
| L 24h | 14.727348487708312 | 128.04500000000002 |
| R 0h | 6.35004578395268 | 77.73 |
| R 3h | 8.706981103025713 | 85.2 |
| R 24h | 4.721774798795134 | 58.14000000000001 |
### Chart
| Category | qRT-PCR | RNA-seq |
|---|---|---|
| L 0h | 1.0266379526770184 | 370.97333333333336 |
| L 3h | 0.033409281988041696 | 19.483333333333334 |
| L 24h | 0.017640642393202205 | 8.74 |
| R 0h | 0.11888857115041833 | 45.63666666666666 |
| R 3h | 0.023405539421417598 | 20.53333333333333 |
| R 24h | 0.00992955653554796 | 6.78 |TRINITY_DN298215_c15_g24
TRINITY_DN295303_c0_g1
TRINITY_DN278631_c1_g1
TRINITY_DN290051_c6_g11
### Chart
| Category | qRT-PCR | RNA-seq |
|---|---|---|
| L 0h | 1.152953859754857 | 0.0 |
| L 3h | 63.93829751520125 | 2.31 |
| L 24h | 8.384483446818072 | 0.5366666666666666 |
| R 0h | 131.36433213179632 | 1.3166666666666667 |
| R 3h | 662.7491375191815 | 16.573333333333334 |
| R 24h | 122.74542094379272 | 2.61 |
### Chart
| Category | qRT-PCR | RNA-seq |
|---|---|---|
| L 0h | 1.0037500696944586 | 13.475 |
| L 3h | 2.06921671991232 | 9.366666666666665 |
| L 24h | 2.1916880333723907 | 5.91 |
| R 0h | 54.78181324352522 | 195.89000000000001 |
| R 3h | 60.677169006637094 | 347.95666666666665 |
| R 24h | 49.26868056602584 | 226.87666666666667 |
### Chart
| Category | qRT-PCR | RNA-seq |
|---|---|---|
| L 0h | 1.0079180431527806 | 8.043333333333333 |
| L 3h | 0.2800746876273246 | 4.093333333333333 |
| L 24h | 0.5567149367195816 | 6.64 |
| R 0h | 0.034501284208591144 | 1.2133333333333334 |
| R 3h | 0.01692523732783797 | 0.5933333333333333 |
| R 24h | 0.007778564850682304 | 0.19999999999999998 |
### Chart
| Category | qRT-PCR | RNA-seq |
|---|---|---|
| L 0h | 1.0071816027611662 | 1.0599999999999998 |
| L 3h | 0.7161625804309809 | 0.29333333333333333 |
| L 24h | 1.2115432758922322 | 0.5666666666666668 |
| R 0h | 0.8160971606539489 | 11.666666666666666 |
| R 3h | 2.989366800125706 | 411.4666666666667 |
| R 24h | 1.3552161534925524 | 86.19333333333333 |TRINITY_DN283339_c1_g1
TRINITY_DN296020_c1_g4
TRINITY_DN279875_c1_g2
TRINITY_DN295792_c0_g1
### Chart
| Category | qRT-PCR | RNA-seq |
|---|---|---|
| L 0h | 1.007616539376585 | 2.7533333333333334 |
| L 3h | 1.4870567479463919 | 4.066666666666666 |
| L 24h | 2.6408740066946605 | 13.450000000000001 |
| R 0h | 0.8273572274529054 | 3.4233333333333333 |
| R 3h | 0.4923202756769562 | 0.9466666666666667 |
| R 24h | 0.6478238648253495 | 1.54 |
### Chart
| Category | qRT-PCR | RNA-seq |
|---|---|---|
| L 0h | 1.0024547313384897 | 17.52 |
| L 3h | 0.392385421828366 | 7.103333333333334 |
| L 24h | 0.5311870801113866 | 11.530000000000001 |
| R 0h | 0.17128495506656286 | 0.11 |
| R 3h | 0.2427779544759966 | 1.2699999999999998 |
| R 24h | 0.44186253999352787 | 0.16 |
### Chart
| Category | qRT-PCR | RNA-seq |
|---|---|---|
| L 0h | 1.0056951771035247 | 0.0 |
| L 3h | 0.6582235546292822 | 0.2333333333333333 |
| L 24h | 4.094979195393211 | 0.24333333333333332 |
| R 0h | 0.5397905940373632 | 0.06999999999999999 |
| R 3h | 18.133516354810627 | 1.7733333333333334 |
| R 24h | 0.9175323473762251 | 0.31333333333333335 |
### Chart
| Category | qRT-PCR | RNA-seq |
|---|---|---|
| L 0h | 1.0140348325332271 | 0.9800000000000001 |
| L 3h | 33.260664274808036 | 40.84 |
| L 24h | 7.490480115422524 | 14.340000000000002 |
| R 0h | 0.27757002576190276 | 0.9400000000000001 |
| R 3h | 0.3004969239058871 | 1.7433333333333334 |
| R 24h | 0.10868238573067536 | 0.1366666666666667 |TRINITY_DN289673_c0_g1
TRINITY_DN278846_c1_g1
TRINITY_DN193494_c0_g1
### Chart
| Category | qRT-PCR | RNA-seq |
|---|---|---|
| L 0h | 0.0 | 0.11333333333333333 |
| L 3h | 0.0 | 0.0 |
| L 24h | 0.0 | 0.0 |
| R 0h | 1.0007617666792428 | 53.376666666666665 |
| R 3h | 0.003409313266289895 | 0.02 |
| R 24h | 0.005139482749221965 | 0.0 |
### Chart
| Category | qRT-PCR | RNA-seq |
|---|---|---|
| L 0h | 0.9985147212905233 | 0.03333333333333333 |
| L 3h | 1.8937438383764953 | 0.5 |
| L 24h | 3.15657859137766 | 1.3333333333333333 |
| R 0h | 1.0682337279267478 | 0.0 |
| R 3h | 17.06894714073817 | 1.04 |
| R 24h | 24.608630822474506 | 5.946666666666666 |
### Chart
| Category | qRT-PCR | RNA-seq |
|---|---|---|
| L 0h | 1.0020001861854089 | 54.803333333333335 |
| L 3h | 3.046450063050869 | 227.78 |
| L 24h | 2.4425461421267616 | 178.09666666666666 |
| R 0h | 0.7324051099232497 | 29.02333333333333 |
| R 3h | 0.7569809218432453 | 31.356666666666666 |
| R 24h | 0.3427959875219104 | 21.73 |Figure S2. Expression of the selected 15 genes inferred at T0, T3 and T24 by RNA-seq and qRT-PCR. L and R represented leaves and roots, respectively. The red lines and black columns represented RNA-seq value and qPCR data, respectively. Error bars represent standard deviation (n = 3).

## Slide 3
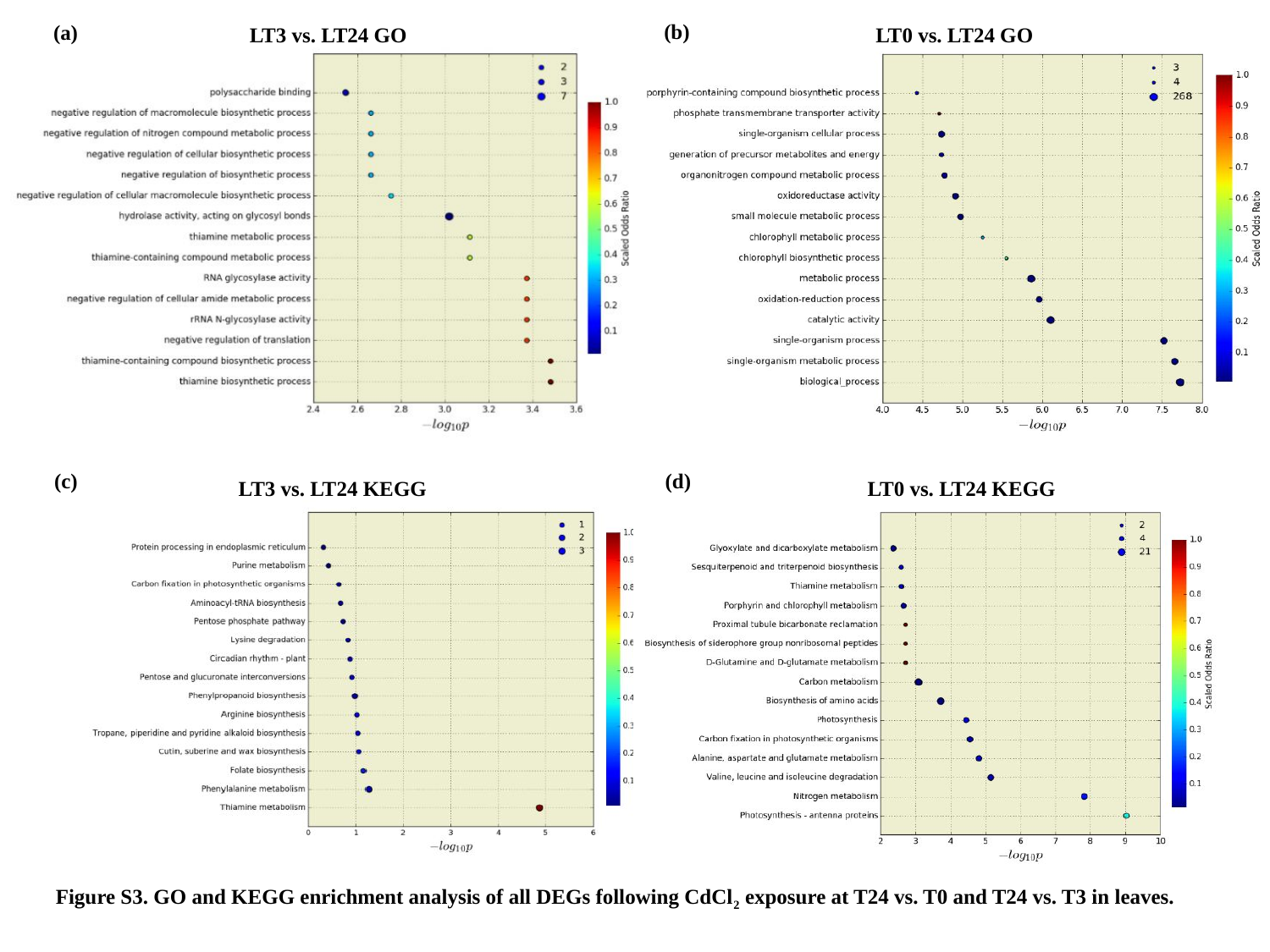

(b)
(a)
LT0 vs. LT24 GO
LT3 vs. LT24 GO
(d)
(c)
LT3 vs. LT24 KEGG
LT0 vs. LT24 KEGG
Supplementary Fig. 2
Figure S3. GO and KEGG enrichment analysis of all DEGs following CdCl2 exposure at T24 vs. T0 and T24 vs. T3 in leaves.

## Slide 4
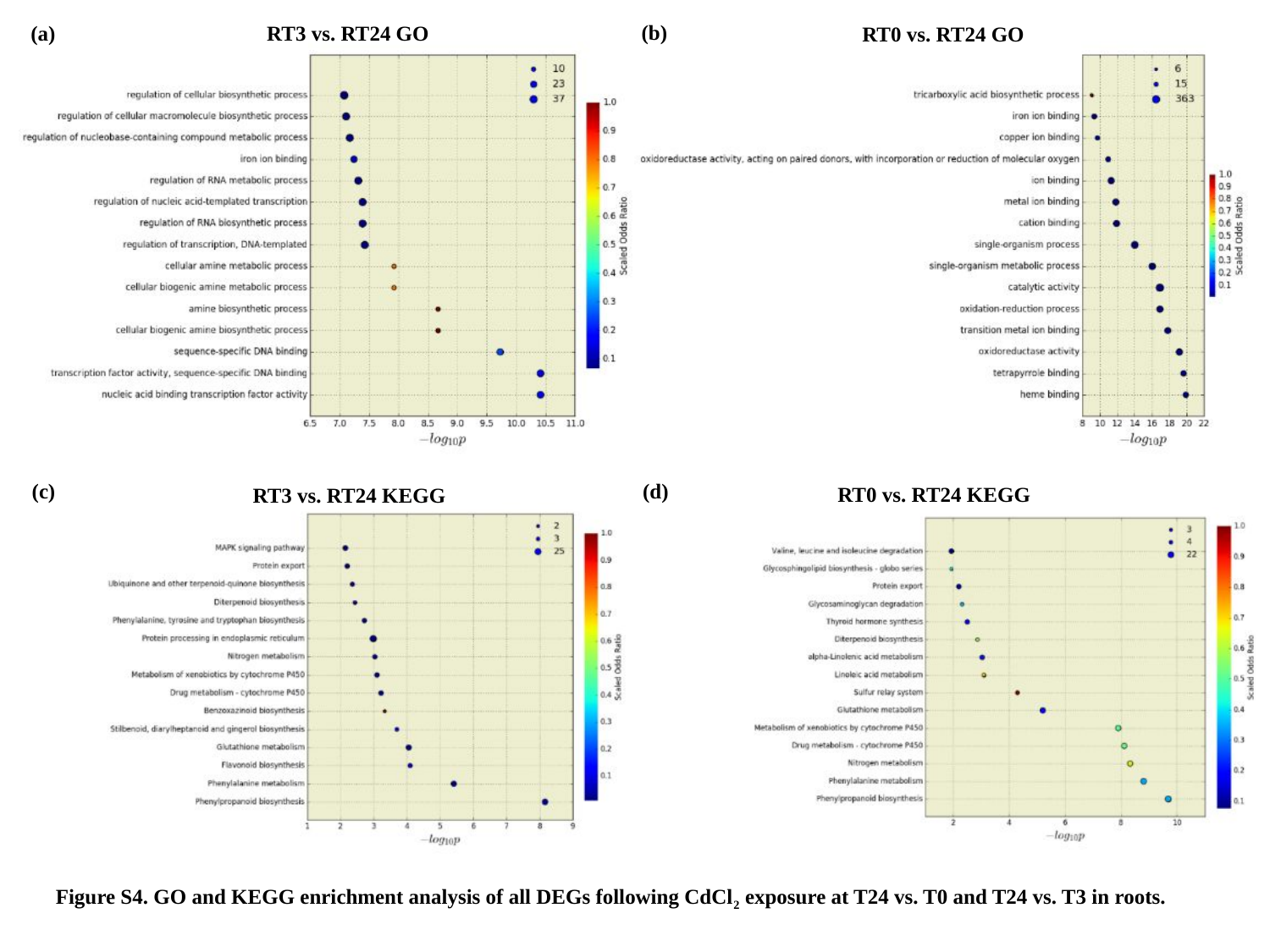

(b)
(a)
RT3 vs. RT24 GO
RT0 vs. RT24 GO
(d)
(c)
RT0 vs. RT24 KEGG
RT3 vs. RT24 KEGG
Figure S4. GO and KEGG enrichment analysis of all DEGs following CdCl2 exposure at T24 vs. T0 and T24 vs. T3 in roots.
